# Impact of Season and Other Factors on Initiation, Discontinuation, and Switching of Systemic Drug Therapy in Patients with Psoriasis: A Retrospective Study

**DOI:** 10.1016/j.xjidi.2022.100171

**Published:** 2022-11-17

**Authors:** Huifang Liang, Brenna Kirk, Jennifer M. Polinski, Xiaomeng Yue, Ryan D. Kilpatrick, Joel M. Gelfand

**Affiliations:** 1 Global Epidemiology, Pharmacovigilance and Patient Safety, AbbVie, North Chicago, Illinois, USA; 2 Aetion, Boston, Massachusetts, USA; 3 Department of Dermatology, University of Pennsylvania Perelman School of Medicine, Philadelphia, Pennsylvania, USA; 4 Department of Biostatistics, Epidemiology & Informatics, Perelman School of Medicine, University of Pennsylvania, Philadelphia, Pennsylvania, USA; 5 Center for Clinical Epidemiology and Biostatistics, Perelman School of Medicine, University of Pennsylvania, Philadelphia, Pennsylvania, USA

**Keywords:** CED, cohort entry date, CI, confidence interval, ICD, International Classification of Diseases, PsA, psoriatic arthritis, RR, relative rate, US, United States

## Abstract

This study investigated whether systemic drug prescribing for psoriasis varies by season and other exacerbating factors. Eligible patients with psoriasis were assessed for each season for initiation, discontinuation, and switching of systemic drugs. A total of 360,787 patients were at risk of initiating any systemic drugs in 2016‒2019; 39,572 patients and 35,388 patients were at risk of drug discontinuation or switching to a biologic and a nonbiologic systemic drug, respectively. The initiation of biologic therapy in 2016‒2019 peaked in spring (1.28%), followed by summer (1.11%), fall (1.08%), and winter (1.01%). Nonbiologic systemic drugs followed a similar pattern. Those aged 30‒39 years, male, those with psoriatic arthritis, those who live in the South region, those who live in areas with lower altitudes, and those who live in areas with lower humidity had higher initiation with the same seasonality pattern. Discontinuation of biologic drugs peaked in summer, and switching of biologics was highest in spring. Season is associated with initiation, discontinuation, and switching, although seasonality pattern is less clear for nonbiologic systemic drugs. Approximately 14,280 more patients with psoriasis in the United States are estimated to initiate a biologic in spring than in other seasons, and over 840 more biologic users switched in spring than in winter. The findings may provide evidence for healthcare resource planning in psoriasis management.

## Introduction

Psoriasis is a chronic systemic inflammatory disease affecting over 7.5 million adults and 0.9 million children in the United States (US) (Armstrong et al., 2021; Paller et al., 2018). The common symptoms of psoriasis include scaling of the skin, itching, and erythema (Dubertret et al., 2006; Feldman et al., 2017; Pariser et al., 2016). Important comorbidities are cardiometabolic disease, psoriatic arthritis (PsA), gastrointestinal disease, kidney disease, malignancies, infections, and mood disorders (Egeberg, 2016; Takeshita et al., 2017). Psoriasis is associated with reduced work productivity and QOL, which are more affected by increasing psoriasis severity (Villacorta et al., 2020; Korman et al., 2016, 2015). The annual US cost of psoriasis amounted to approximately $112 billion in 2013 (Brezinski et al., 2015). Those with comorbidities are associated with a higher economic burden (Feldman et al., 2017).

Several factors have been shown to contribute to psoriasis flare. These include obesity/weight gain (Aune et al., 2018; Wolk et al., 2009), tobacco smoking (Aune et al., 2018; Wolk et al., 2009), infections (Skudutyte-Rysstad et al., 2014), stress (Chandran and Raychaudhuri, 2010; Farber and Nall, 1984), and low humidity (Chandran and Raychaudhuri, 2010; Farber and Nall, 1984). Few studies have examined seasonality in psoriasis flare (Harvell and Selig, 2016; Kardeş, 2019; Wu et al., 2020); however, there are seasonal fluctuations in these known triggers of body weight (Fahey et al., 2020) and cigarette consumption (Dubertret et al., 2006; Momperousse et al., 2007; Rachakonda et al., 2014). Regional differences have been found in healthcare resource use for psoriasis (Nguyen et al., 2020), psoriasis severity, comorbidities, and treatment response in the US (Enos et al., 2021), and increasing latitude is associated with increased prevalence (Gutierrez et al., 2017; Springate et al., 2017). Over 17% of patients with psoriasis are treated with systemic therapy or phototherapy, an indicator of higher levels of disease severity (Crown et al., 2004). The objectives of this study were to explore seasonal patterns in the initiation, discontinuation, and switching from systemic drugs among patients with psoriasis and to assess whether the change in systemic drugs varies by patient characteristics and other potentially exacerbating factors. The study findings may provide real-world evidence for the use of systemic drugs for psoriasis and help with psoriasis management and healthcare resource planning.

## Results

### Patient population

**Figure 1:**
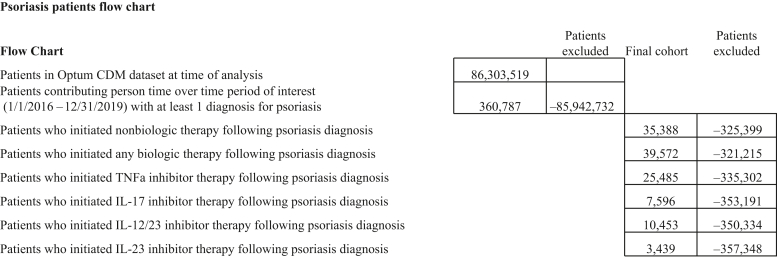
**Patients flow chart.** Source: AETION Evidence Platform.

Patient flow chart for each cohort is presented in Figure 1. A total of 360,787 patients with psoriasis were at risk of initiating any systemic drugs in 2016‒2019 (see Table 1). They were on average aged 54.2 years (SD = 18.2), 52.5% were female, and 6.4% had PsA at cohort entry. The South region of the US had the most patients (44.9%), followed by the Midwest (22.3%), the West (19.1%), and the Northeast regions (13.4%). Two thirds came from latitude ≥39.85 °N; over 60% were from states with average annual relative humidity less than 77.1%. A total of 39,572 patients were at risk of discontinuing or switching from any biologics (see Table 1). They consisted of patients who initiated biologics or continued their existing biologics. They were younger (mean age 48.9 vs. 56.6 years) and more likely to be male (52.8 vs. 43.4%) than the 35,388 patients who were at risk of discontinuing or switching from any nonbiologic systemic drugs. Among biologic users, the ratio of patients who were at risk of discontinuing or switching from TNF-α inhibitors, IL-12/23 inhibitors, IL-17 inhibitors, and IL-23 inhibitors was roughly 7:3:2:1, highest for those who entered the market earliest, lowest for the newest drug class.

**Table 1:** Demographics and Characteristics of Patients with Psoriasis on Systemic Therapy in 2016‒2019

Characteristics		At Risk of Discontinuing or Switching from					
	At Risk of Initiation	Nonbiologic Systemic Therapy	Any Biologic Therapy	TNF-α Inhibitor	IL-17 Inhibitors	IL-12/23 Inhibitors	IL-23 Inhibitors
n	360,787	35,388	39,572	25,485	7,596	10,453	3,439
Age (y)							
Mean (SD)	54.2 (18.2)	56.6 (15.3)	48.9 (14.2)	49.4 (14.3)	50.6 (13.2)	47.7 (14.3)	47.9 (13.4)
<20	13,720 (3.8)	414 (1.2)	776 (2.0)	526 (2.1)	40 (0.5)	270 (2.6)	37 (1.1)
20‒29	25,016 (7.0)	1,465 (4.2)	3,138 (7.9)	1,878 (7.4)	427 (5.6)	930 (8.9)	297 (8.6)
30‒39	42,605 (11.8)	3,404 (9.6)	6,688 (16.9)	4,088 (16.0)	1,199 (15.8)	1,904 (18.2)	639 (18.6)
40‒49	54,705 (15.2)	5,518 (15.6)	8,947 (22.6)	5,663 (22.2)	1,774 (23.4)	2,425 (23.2)	857 (24.9)
50‒59	68,905 (19.2 )	8,233 (23.3)	10,561 (26.7)	6,838 (26.8)	2,194 (28.9)	2,723 (26.0)	904 (26.3)
60‒69	74,811 (20.8)	8,692 (24.6)	6,860 (17.3)	4,692 (18.4)	1,375 (18.1)	1,591 (15.2)	545 (15.8)
≥70	79,983 (22.2)	7,555 (21.4)	2,597 (6.6)	1,795 (7.0)	587 (7.7)	610 (5.8)	160 (4.7)
Missing	1,042	7	5	5	0	0	0
Sex							
Male	170,946 (47.4)	15,301 (43.4)	20,913 (52.9)	13,428 (52.7)	3,823 (50.3)	5,419 (51.8)	1,893 (55.0)
Female	189,545 (52.6)	19,984 (56.6)	18,656 (47.1)	12,054 (47.3)	3,773 (49.7)	5,034 (48.2)	1,546 (45.0)
Unknown/missing	296	3	3	3	0	0	0
Region							
Northeast	48,413 (13.5)	3,847 (10.9)	3,940 (10.0)	2,349 (9.2)	666 (8.8)	1,225 (11.7)	384 (11.2)
Midwest	80,595 (22.4)	7,969 (22.6)	9,223 (23.3)	6,234 (24.5)	1,686 (22.2)	2,344 (22.5)	696 (20. 3)
South	162,079 (45.0)	16,895 (47.9)	19,848 (50.2)	12,705 (49.9)	3,971 (52.3)	5,062 (48.5)	1,795 (52.2)
West	68,829 (19.1)	6,528 (18.5)	6,509 (16.5)	4,161 (16.4)	1,268 (16.7)	1,807 (17.3)	562 (16.4)
Other/missing	871	49	52	36	5	15	2
Humidity							
≥77.1%	138,633 (38.5)	13,079 (37.1)	14,354 (36.3)	9,391 (36.9)	2,623 (34.5)	3,865 (37.0)	1,185 (34.5)
<77.1%	221,283 (61.5)	22,175 (62.9)	25,188 (63.7)	16,074 (63.1)	4,969 (65.5)	6,577 (63.0)	2,253 (65.5)
Missing	871	34	30	20	4	11	1
Latitude							
<39.85 °N	238,273 (66.2)	24,105 (68.4)	27,175 (68.7)	17,434 (68.5)	5,372 (70.8)	7,068 (67.7)	2,394 (69.6)
≥39.85 °N	121,652 (33.8)	11,149 (31.6)	12,367 (31.3)	8,031 (31.5)	2,220 (29.2)	3,374 (32.3)	1,044 (30.4)
Missing	862	34	30	20	4	11	1
Psoriatic arthritis							
Yes	23,212 (6.4)	11,694 (33.1)	14,350 (36.3)	10,918 (42.8)	3,454 (45.5)	2,919 (27.9)	821 (23.9)
No	337,575 (93.7)	23,694 (79.9)	25,222 (63.7)	14,467 (57.2)	4142 (54.5)	7,534 (72.1)	2,618 (76.1)

Note: Data are presented as frequency (percentage) unless otherwise specified.

### Impact of season on initiation of systemic drugs

**Figure 2:**
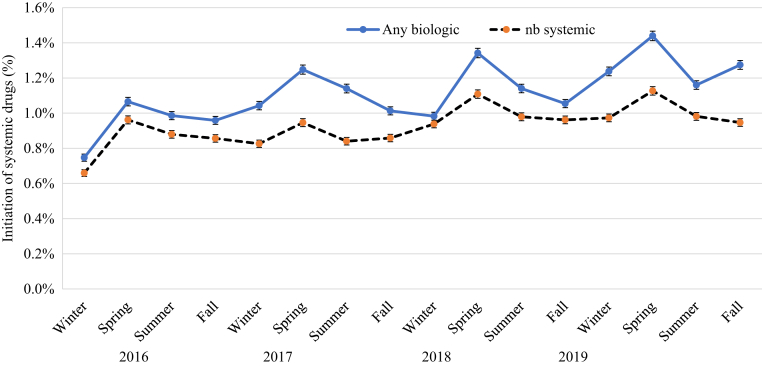
**The impact of season on the initiation of systemic drugs in patients with psoriasis in Optum claims databases in 2016‒2019.** nb, nonbiologic.

**Figure 3:**
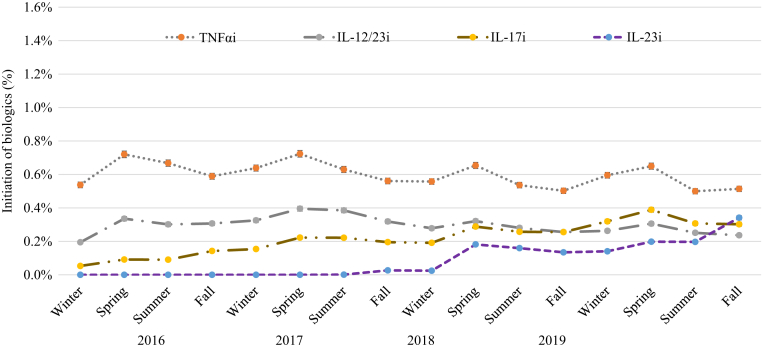
**The impact of season on the initiation of systemic drugs by biologic drug class in patients with psoriasis in 2016‒2019.** IL-12/23i, IL-12/23 inhibitor; IL-17i, IL-17 inhibitor; IL-23i, IL-23 inhibitor; TNFαi, TNF-α inhibitor.

Figure 2 shows the impact of season on the initiation of systemic drugs in patients with psoriasis in 2016‒2019. Initiation of biologics is on average higher than that of nonbiologic systemic drugs (0.9‒1.4% vs. 0.8‒1.1%). The initiation of nonbiologic systemic drugs peaked in spring (0.9‒1.1%). The initiation of any biologic followed a pattern similar to that of nonbiologic systemic drugs, with the incidence highest in spring (ranging from 1.1 to 1.4%) and lower in other seasons (ranging from 1.0 to 1.3%). Table 2 presents the incidence and 95% confidence interval (CI) of initiation of biologic therapy and stratified by different factors in patients with psoriasis in 2016‒2019. The mean incidence of initiation of biologic therapy (95% CI) in 2016‒2019 was highest in spring (1.28% [1.25‒1.30%]), followed by that in summer (1.11% [1.08‒1.13%]), fall (1.08% [1.06‒1.10%]), and winter (1.01% [0.99‒1.03%]). Figure 3 shows that biologics initiation by drug class also peaked in spring. Among biologics, the incidence of initiation seemed to be highest for TNF-α inhibitors, followed by that in IL-12/IL-23 inhibitors, IL-17 inhibitors, and IL-23 inhibitors. To account for the clustering within the data due to repeated measures in Table 2, the meta-regression analysis results in Table 3 confirms that the incidence of initiation of biologics was the highest in spring among the four seasons. The incidence of initiating biologics overall was 13% lower in summer (relative rate [RR] = 0.87, 95% CI = 0.82–0.92), 16% lower in fall (RR = 0.84, 95% CI = 0.84–0.95), and 21% lower in winter (RR = 0.79, 95% CI = 0.69–0.91) than in spring. The initiation trend was consistent by biologic class and patient characteristics (sex, age, and, PsA status).

**Table 2:** Incidence (%) and 95% Confidence Intervals of Initiation of Biologic Therapy Stratified by Different Factors in Patients with Psoriasis in 2016‒2019

Factors	Winter, 2016‒2019	Spring, 2016‒2019	Summer, 2016‒2019	Fall, 2016‒2019	Mean, 2016–2019
All	1.01 (0.99**‒**1.03)	1.28 (1.25**‒**1.30)	1.11 (1.08**‒**1.13)	1.08 (1.06**‒**1.10)	1.12 (1.10**‒**1.14)
Biologic class					
TNF-α inhibitors	0.58 (0.57**‒**0.60)	0.69 (0.67**‒**0.70)	0.58 (0.56**‒**0.60)	0.54 (0.52**‒**0.56)	0.60 (0.58**‒**0.61)
IL-17 inhibitors	0.18 (0.17**‒**0.19)	0.25 (0.24**‒**0.26)	0.22 (0.21**‒**0.23)	0.23 (0.22**‒**0.24)	0.22 (0.21**‒**0.23)
IL-12/23 inhibitors	0.27 (0.25**‒**0.28)	0.34 (0.33**‒**0.35)	0.30 (0.29**‒**0.32)	0.28 (0.27**‒**0.29)	0.30 (0.28**‒**0.31)
IL-23 inhibitors	0.08 (0.07**‒**0.09)	0.19 (0.18**‒**0.20)	0.12 (0.11**‒**0.13)	0.17 (0.16**‒**0.18)	0.14 (0.13**‒**0.15)
Sex					
Male	1.16 (1.12**‒**1.19)	1.45 (1.41**‒**1.49)	1.25 (1.21**‒**1.29)	1.23 (1.20**‒**1.27)	1.27 (1.2**‒**1.31)
Female	0.88 (0.85**‒**0.91)	1.12 (1.09**‒**1.15)	0.98 (0.95**‒**1.01)	0.94 (0.91**‒**0.97)	0.98 (0.9**‒**1.01)
Age (y)					
<20	0.54 (0.44**‒**0.63)	0.65 (0.55**‒**0.76)	0.71 (0.60**‒**0.82)	0.72 (0.60**‒**0.83)	0.65 (0.5**‒**0.76)
20‒29	1.41 (1.29**‒**1.54)	1.97 (1.83**‒**2.12)	1.72 (1.59**‒**1.86)	1.65 (1.52**‒**1.79)	1.69 (1.6**‒**1.82)
30‒39	1.83 (1.73**‒**1.93)	2.51 (2.39**‒**2.63)	2.07 (1.97**‒**2.18)	2.09 (1.98**‒**2.20)	2.12 (2.0**‒**2.23)
40‒49	1.76 (1.68**‒**1.85)	2.27 (2.18**‒**2.37)	2.01 (1.92**‒**2.10)	1.98 (1.89**‒**2.07)	2.00 (1.9**‒**2.09)
50‒59	1.59 (1.52**‒**1.66)	1.96 (1.88**‒**2.04)	1.72 (1.65**‒**1.79)	1.69 (1.62**‒**1.76)	1.74 (1.7**‒**1.81)
60‒69	0.90 (0.85**‒**0.94)	1.11 (1.05**‒**1.16)	0.95 (0.90**‒**1.00)	1.00 (0.95**‒**1.05)	0.99 (0.9**‒**1.04)
≥70	0.22 (0.21**‒**0.24)	0.27 (0.25**‒**0.29)	0.23 (0.21**‒**0.25)	0.24 (0.22**‒**0.25)	0.24 (0.2**‒**0.26)
PsA					
Yes	4.25 (4.10**‒**4.39)	5.10 (4.94**‒**5.26)	4.47 (4.32**‒**4.61)	4.19 (4.05**‒**4.33)	4.49 (4.3**‒**4.64)
No	0.78 (0.76**‒**0.80)	0.99 (0.96**‒**1.01)	0.85 (0.83**‒**0.87)	0.84 (0.82**‒**0.86)	0.86 (0.8**‒**0.89)
Region					
Northeast	0.71 (0.65**‒**0.76)	0.89 (0.83**‒**0.95)	0.87 (0.81**‒**0.93)	0.80 (0.74**‒**0.85)	0.82 (0.8**‒**0.87)
Midwest	1.14 (1.08**‒**1.19)	1.30 (1.25**‒**1.36)	1.11 (1.06**‒**1.16)	1.10 (1.05**‒**1.15)	1.16 (1.1**‒**1.21)
South	1.16 (1.12**‒**1.20)	1.52 (1.48**‒**1.56)	1.30 (1.26**‒**1.34)	1.25 (1.21**‒**1.29)	1.31 (1.3**‒**1.35)
West	0.76 (0.72**‒**0.80)	1.00 (0.95**‒**1.04)	0.86 (0.82**‒**0.91)	0.88 (0.84**‒**0.93)	0.88 (0.8**‒**0.92)
Latitude					
≥39.85 °N	0.93 (0.88**‒**0.97)	0.95 (0.92**‒**0.99)	1.12 (1.07**‒**1.16)	0.98 (0.95**‒**1.02)	1.00 (1.0**‒**1.04)
<39.85 °N	1.13 (1.10**‒**1.17)	1.04 (1.01**‒**1.07)	1.36 (1.33**‒**1.39)	1.17 (1.14**‒**1.20)	1.18 (1.1**‒**1.21)
Humidity					
≥77.1%	0.93 (0.89**‒**0.97)	0.94 (0.91**‒**0.97)	1.12 (1.08**‒**1.15)	0.97 (0.94**‒**1.01)	0.99 (1.0**‒**1.03)
<77.1%	1.15 (1.11**‒**1.19)	1.06 (1.03**‒**1.09)	1.39 (1.35**‒**1.42)	1.20 (1.17**‒**1.23)	1.20 (1.2**‒**1.23)

Abbreviation: PsA, psoriatic arthritis.

**Table 3:** Relative Risk and 95% CI for Initiation of Biologic Therapy: Meta-Regression Analysis Using Robust Variance Estimation Method and Aggregate Data from 16 Seasons in 2016‒2019

Factors	Winter Vs Spring	Summer Vs Spring	Fall Vs Spring
All	0.79 (0.69–0.91)	0.87 (0.82–0.92)	0.84 (0.84–0.85)
Biologic class			
TNF-αi	0.85 (0.83–0.87)	0.85 (0.8–0.9)	0.79 (0.78–0.79)
IL-17i	0.75 (0.59–0.96)	0.83 (0.77–0.9)	0.82 (0.76–0.9)
IL-12/23i	0.78 (0.67–0.92)	0.9 (0.81–0.99)	0.82 (0.8–0.84)
IL-23i	0.32 (0.25–0.39)	0.23 (0.01–5.9)	0.58 (0.44–0.75)
Sex			
Male	0.78 (0.78–0.78)	0.86 (0.86–0.86)	0.85 (0.85–0.85)
Female	0.78 (0.71–0.87)	0.88 (0.83–0.93)	0.84 (0.83–0.85)
Age (y)			
<20	0.83 (0.44–1.58)	1.08 (1.05–1.1)	1.1 (1.08–1.12)
20–29	NA	NA	NA
30–39	0.72 (0.13–4.02)	0.83 (0.31–2.19)	0.83 (0.25–2.71)
40–49	0.77 (0.71–0.85)	0.88 (0.8–0.97)	0.87 (0.86–0.88)
50–59	0.8 (0.55–1.16)	0.88 (0.43–1.8)	0.86 (0.51–1.46)
60–69	NA	NA	NA
≥70	0.81 (0.49–1.34)	NA	NA
PsA			
Yes	0.83 (0.76–0.91)	0.88 (0.85–0.9)	0.82 (0.81–0.83)
No	0.79 (0.7–0.9)	0.86 (0.81–0.93)	0.86 (0.85–0.86)
Region			
Northeast	NA	NA	NA
Midwest	NA	NA	NA
South	0.76 (0.04–13.6)	NA	NA
West	NA	NA	NA
Latitude			
≥39.85 °N	NA	NA	NA
<39.85 °N	0.76 (0.7–0.83)	0.86 (0.83–0.9)	0.84 (0.83–0.84)
Humidity			
≥77.1%	NA	0.87 (0.7–1.09)	0.86 (0.53–1.4)
<77.1%	0.76 (0.7–0.83)	0.87 (0.82–0.92)	0.83 (0.82–0.85)

Abbreviations: CI, confidence interval; IL-17i, IL-17 inhibitor; IL-23i, IL-23 inhibitor; TNFαi, TNF-α inhibitor; NA, not applicable; IL-12/23i, IL-12/23 inhibitor.

Note: Robust variance estimation was used to estimate the covariance matrix of the correlated coefficients in the meta-regression accounting for the clustering within the data owing to repeated measures (Fisher et al., 2017). The meta-regression model was fitted with the incidence transformed into a natural logarithmic scale and season as a fixed effect. For IL-17 inhibitors, only the data from 2018 and 2019 are used, to minimize the effect of multiple new products launched in 2015 and 2016.

**Figure 4:**
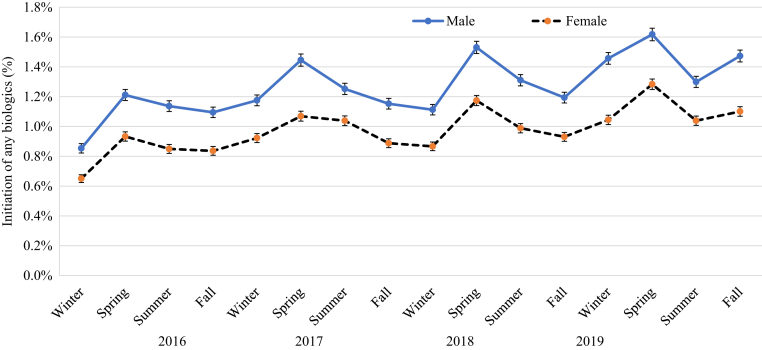
**The impact of season on the initiation of biologics stratified by sex in patients with psoriasis in 2016‒2019**.

**Figure 5:**
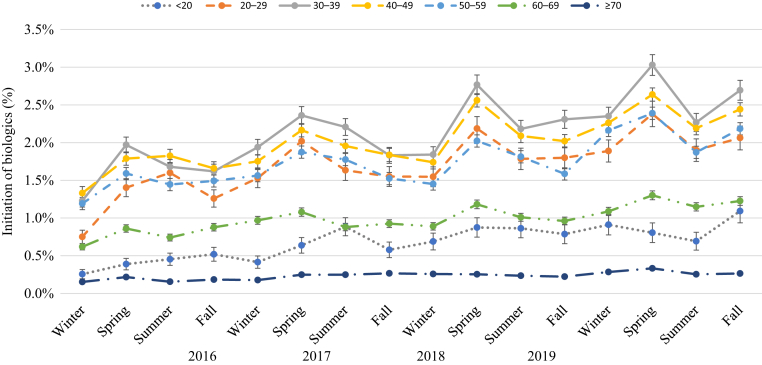
**The impact of season on the initiation of biologics stratified by age in patients with psoriasis in 2016‒2019**.

**Figure 6:**
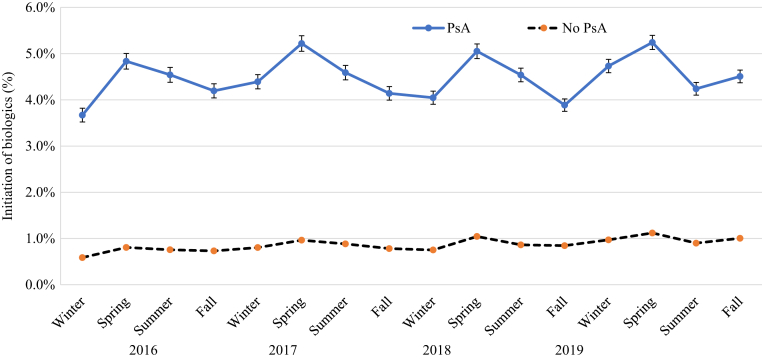
**The impact of season on the initiation of biologics stratified by psoriatic arthritis in patients with psoriasis in 2016‒2019.** PsA, psoriatic arthritis.

**Figure 7:**
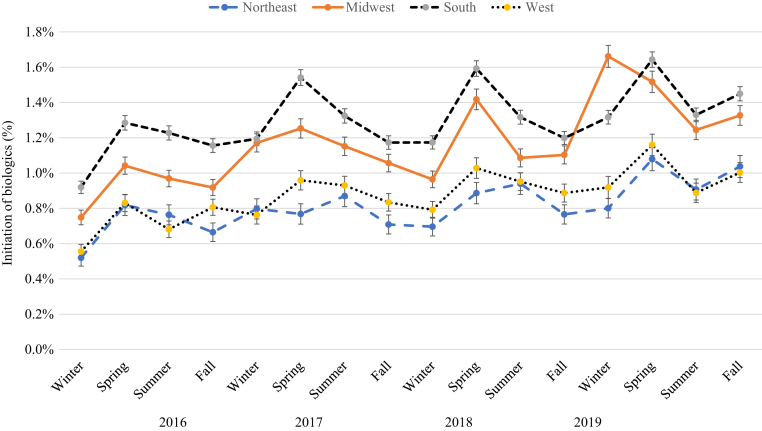
**The impact of season on the initiation of biologics stratified by region in patients with psoriasis in 2016‒2019**.

**Figure 8:**
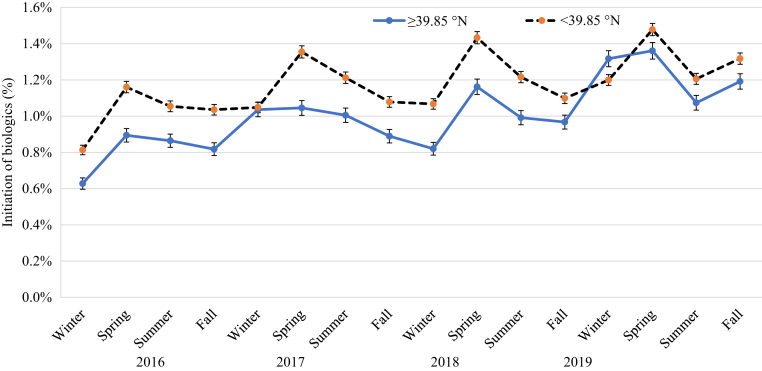
**The impact of season on the initiation of biologics stratified by latitude in patients with psoriasis in 2016‒2019**.

**Figure 9:**
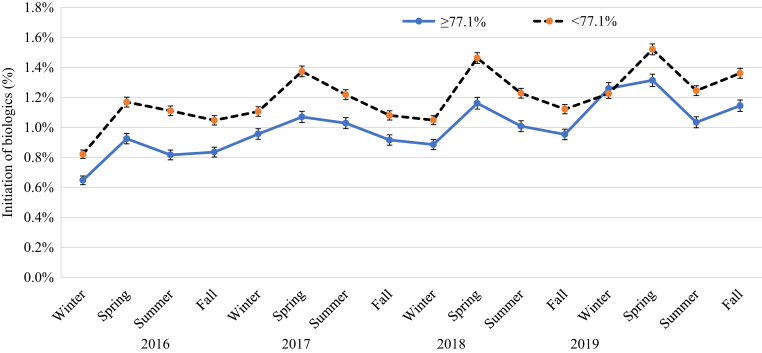
**The impact of season on the initiation of biologics stratified by humidity in patients with psoriasis in 2016‒2019**.

Figure 4, Figure 5, Figure 6, Figure 7, Figure 8, Figure 9 show that when stratified by sex; age; diagnosis of PsA; and US region, latitude, and humidity, the peak in the initiation of any biologic therapy was spring, followed by summer and fall or winter. The mean incidence of initiation of systemic drugs was higher in males than in females in all seasons (1.16‒1.45% in males vs. 0.88‒1.12% in females), with seasonal differences quite similar in males and females. When stratified by age, the incidence of initiation of systemic drugs was highest in those aged 30‒39 years, followed by those 40‒49 years, 20‒29 years, 50‒59 years, 60‒69 years, <20 years, and ≥70 years. The seasonal pattern is very clear in all age subgroups except in those aged <20 years, who had higher initiation in summer and fall. Patients with PsA are at least five times more likely to initiate any biologics than those without (mean incidence = 4.19‒5.10% in PsA vs. 0.78‒0.99% in non-PsA). The seasonal pattern is more obvious in patients with PsA than in those without, with seasonal differences ranging from 0.3 to 1.2% in PsA and from 0.1 to 0.2% in those without PsA. The incidence of any biologic initiation appeared to be higher in patients in the South and Midwest regions than in those in the West and Northeast regions (mean incidence = 1.16‒1.52% in the South vs. 1.10‒1.30% in the Midwest vs. 0.76‒1.00% in the West vs. 0.71‒0.89% in the Northeast). Initiation of biologics appeared to be higher in latitude <39.85 °N than in latitude ≥39.85 °N (1.0‒1.5% vs. 0.8‒1.4%) and in lower (<77.1%) versus higher (≥77.1%) humidity regions.

### Impact of season on discontinuation of systemic drugs

**Figure 10:**
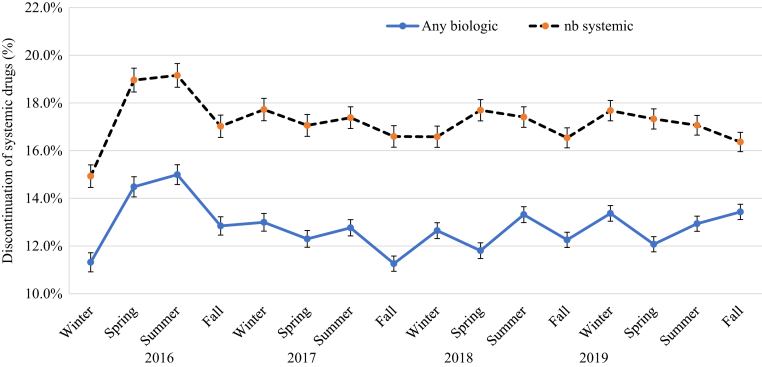
**The impact of season on the discontinuation of systemic drugs in patients with psoriasis in 2016‒2019.** nb, nonbiologic.

Figure 10 shows the impact of season on discontinuation of systemic drugs in patients with psoriasis in 2016‒2019. The incidence of discontinuation was lower for biologics (ranging from 11.3 to 15.0%) than for nonbiologic systemic drugs (ranging from 14.9 to 19.2%) across seasons, with the incidence difference between nonbiologic systemic drugs and biologics, largest in spring. The peak of discontinuation was inconsistent for biologics or nonbiologic systemic drugs; discontinuation peaked in two winters for nonbiologic systemic drugs and in two summers for biologics. Further analysis of biologic discontinuation by drug class based on a four-season mean showed that discontinuation for TNF-α inhibitors and IL-17 inhibitors appeared to be highest in winter and that discontinuation for IL-12/IL-23 inhibitors appeared to be highest in summer (see Table 4). The mean incidence of discontinuation of biologics seemed to be highest in summer overall and in stratified analyses, except for patients aged ≥60 years and patients with PsA, who had the highest discontinuation rate in the winter (see Table 4). The incidence of discontinuation of biologic drugs does not seem to differ by sex but seems lower in patients with PsA, patients in the West and Midwest regions, patients in latitude ≥39.85 °N, and patients in higher humidity regions. In addition, the incidence of discontinuation of biologics among adults aged ≥20 years decreases with increasing age, lowest in those who were aged ≥70 years (see Table 4). For nonbiologic systemic therapy, both the mean incidence of discontinuation and stratified analyses seemed to be higher in spring and summer (data not shown). In Table 5, the results from meta-regression analysis show that the incidence of discontinuation of biologics overall was 7% higher in summer than in spring (RR = 1.07, 95% CI = 1.07–1.07) but was not significantly different in fall (RR = 0.99, 95% CI = 0.93–1.06) and winter (RR = 1.00, 95% CI = 0.90–1.12). The discontinuation trend was consistent by biologic class and patient characteristics (sex, age, and PsA status).

**Table 4:** Incidence (%) and 95% Confidence Intervals of Discontinuation of Biologic Therapy Stratified by Different Factors in Patients with Psoriasis in 2016‒2019

Factors	Winter, 2016–2019	Spring, 2016–2019	Summer, 2016–2019	Fall, 2016–2019	Mean, 2016–2019
All	12.7 (12.4**‒**13.1)	12.5 (12.2**‒**12.9)	13.4 (13.0**‒**13.7)	12.5 (12.1**‒**12.8)	12.8 (12.4**‒**13.1)
Biologic class					
TNF-α inhibitors	9.8 (9.4**‒**10.2)	9.2 (8.9**‒**9.6)	9.4 (9.0**‒**9.8)	9.0 (8.6**‒**9.3)	9.4 (9.0**‒**9.7)
IL-17 inhibitors	10.5 (9.6**‒**11.4)	8.4 (7.6**‒**9.2)	10.1 (9.3**‒**10.8)	9.2 (8.5**‒**9.9)	9.5 (8.7**‒**10.3)
IL-12/23 inhibitors	21.5 (20.5**‒**22.4)	23.1 (22.1**‒**24.1)	23.4 (22.4**‒**24.3)	21.6 (20.6**‒**22.5)	22.4 (21.4**‒**23.3)
Sex					
Male	12.7 (12.2**‒**13.2)	12.5 (12.0**‒**13.0)	13.6 (13.2**‒**14.1)	12.2 (11.8**‒**12.7)	12.8 (12.3**‒**13.2)
Female	12.7 (12.2**‒**13.2)	12.5 (12.0**‒**13.0)	13.1 (12.6**‒**13.6)	12.7 (12.8**‒**13.2)	12.8 (12.3**‒**13.3)
Age (y)					
<20	14.8 (11.5**‒**18.2)	15.3 (12.0**‒**18.6)	18.4 (15.2**‒**21.7)	14.3 (11.1**‒**17.4)	15.8 (12.6**‒**19.1)
20**–**29	19.7 (17.8**‒**21.6)	18.8 (17.0**‒**20.6)	21.7 (20.0**‒**23.5)	19.9 (18.1**‒**21.7)	20.1 (18.3**‒**21.9)
30**–**39	16.0 (14.9**‒**17.0)	16.6 (15.6**‒**17.6)	18.5 (17.5**‒**19.5)	17.6 (16.6**‒**18.7)	17.2 (16.2‒18.3)
40**–**49	14.3 (13.5**‒**15.0)	14.3 (13.5**‒**15.1)	15.5 (14.7**‒**16.3)	14.7 (13.9**‒**15.5)	14.7 (13.9**‒**15.5)
50**–**59	11.6 (10.9**‒**12.2)	11.8 (11.1**‒**12.4)	12.2 (11.5**‒**12.8)	11.7 (11.1**‒**12.3)	11.8 (11.2**‒**12.4)
60**–**69	10.7 (10.0**‒**11.4)	9.2 (8.5**‒**9.8)	9.4 (8.7**‒**10.0)	9.1 (8.5**‒**9.8)	9.6 (8.9**‒**10.2)
≥70	8.8 (7.8**‒**9.7)	8.6 (7.6**‒**9.5)	7.6 (6.7**‒**8.5)	7.1 (6.3**‒**7.8)	7.9 (7.1**‒**8.8)
PsA					
Yes	11.3 (10.9**‒**11.8)	10.7 (10.2**‒**11.2)	10.9 (10.4**‒**11.3)	10.4 (10.0**‒**10.8)	10.8 (10.4**‒**11.3)
No	14.0 (13.6**‒**14.5)	14.0 (13.6**‒**14.5)	15.2 (14.7**‒**15.6)	14.1 (13.7**‒**14.6)	14.3 (13.9**‒**14.8)
Region					
Northeast	13.0 (11.8**‒**14.1)	13.0 (11.8**‒**14.1)	13.3 (12.2**‒**14.4)	12.1 (11.1**‒**13.1)	12.8 (11.7**‒**13.9)
Midwest	12.0 (11.3**‒**12.7)	12.0 (11.3**‒**12.7)	12.5 (11.9**‒**13.2)	12.0 (11.4**‒**12.7)	12.2 (11.5**‒**12.8)
South	13.3 (12.8**‒**13.8)	12.9 (12.4**‒**13.4)	14.2 (13.8**‒**14.7)	13.1 (12.6**‒**13.5)	13.4 (12.9**‒**13.9)
West	11.7 (10.9**‒**12.5)	11.9 (11.1**‒**12.7)	12.1 (11.3**‒**12.8)	11.5 (10.7**‒**12.3)	11.8 (11.0**‒**12.6)
Latitude					
≥39.85 °N	12.2 (11.6**‒**12.8)	12.2 (11.6**‒**12.9)	12.4 (11.8**‒**13.0)	11.9 (11.3**‒**12.4)	12.2 (11.6**‒**12.8)
<39.85 °N	12.9 (12.5**‒**13.3)	12.6 (12.2**‒**13.0)	13.8 (13.4**‒**14.3)	12.7 (12.3**‒**13.1)	13.0 (12.6**‒**13.5)
Humidity					
≥77.1%	11.8 (11.2**‒**12.3)	12.1 (11.5**‒**12.6)	12.4 (11.9**‒**13.0)	11.7 (11.2**‒**12.3)	12.0 (11.5**‒**12.5)
<77.1%	13.2 (12.8**‒**13.7)	12.8 (12.3**‒**13.2)	13.9 (13.5**‒**14.4)	12.9 (12.5**‒**13.3)	13.2 (12.8**‒**13.7)

Abbreviation: PsA, psoriatic arthritis.

**Table 5:** Relative Risk and 95% CI for Discontinuation of Biologic Therapy: Meta-Regression Analysis Using Robust Variance Estimation Method and Aggregate Data from 16 Seasons in 2016‒2019

Factors	Winter Vs Spring	Summer Vs Spring	Fall Vs Spring
All	1.00 (0.90–1.12)	1.07 (1.07–1.07)	0.99 (0.93–1.06)
Biologic class			
TNF-αi	NA	1.02 (0.99–1.04)	0.97 (0.67–1.41)
IL-17i	1.28 (1.27–1.3)	1.20 (1.18–1.22)	1.11 (0.98–1.27)
IL-12/23i	NA	NA	0.91 (0.72–1.16)
IL-23i	1.15 (0.54–2.45)	1.17 (0.74–1.86)	1.02 (0.77–1.35)
Sex			
Male	1.00 (0.87–1.15)	1.09 (1.09–1.09)	0.97 (0.92–1.03)
Female	1.01 (0.93–1.09)	1.04 (1.04–1.04)	1.01 (0.94–1.09)
Age (y)			
<20	NA	NA	0.93 (0.17–4.96)
20–29	NA	NA	NA
30–39	0.96 (0.92–1)	1.11 (1.1–1.12)	1.05 (0.96–1.16)
40–49	0.98 (0.89–1.09)	1.08 (1.04–1.12)	1.02 (0.96–1.08)
50–59	0.97 (0.66–1.43)	1.04 (0.57–1.88)	0.99 (0.75–1.29)
60–69	1.15 (1.1–1.21)	1.02 (0.91–1.16)	0.98 (0.87–1.1)
≥70	0.99 (0.63–1.55)	0.87 (0.75–1.01)	0.82 (0.76–0.87)
PsA			
Yes	1.05 (1.03–1.06)	1.02 (0.59–1.76)	NA
No	0.99 (0.86–1.14)	1.08 (1.07–1.08)	1.00 (0.93–1.08)
Region			
Northeast	NA	1.01 (0.81–1.25)	0.91 (0.74–1.14)
Midwest	NA	NA	NA
South	NA	1.11 (0.95–1.28)	1.01 (0.78–1.3)
West	0.98 (0.93–1.03)	1.01 (1.01–1.02)	0.96 (0.81–1.13)
Latitude			
≥39.85 °N	NA	1.01 (0.7–1.46)	0.95 (0.67–1.36)
<39.85 °N	1.01 (0.94–1.09)	1.09 (1.08–1.11)	1.00 (0.96–1.05)
Humidity			
≥77.1%	0.96 (0.54–1.7)	1.03 (0.59–1.77)	NA
<77.1%	NA	NA	NA

Abbreviations: CI, confidence interval; IL-17i, IL-17 inhibitor; IL-23i, IL-23 inhibitor; TNFαi, TNF-α inhibitor; NA, not applicable; IL-12/23i, IL-12/23 inhibitor.

Note: Robust variance estimation was used to estimate the covariance matrix of the correlated coefficients in the meta-regression accounting for the clustering within the data due to repeated measures (Fisher et al., 2017). The meta-regression model was fitted with the incidence transformed into a natural logarithmic scale and season as a fixed effect. For IL-17 inhibitors, only the data from 2018 and 2019 are used, to minimize the effect of multiple new products launched in 2015 and 2016.

### Impact of season on switching of systemic drugs

**Figure 11:**
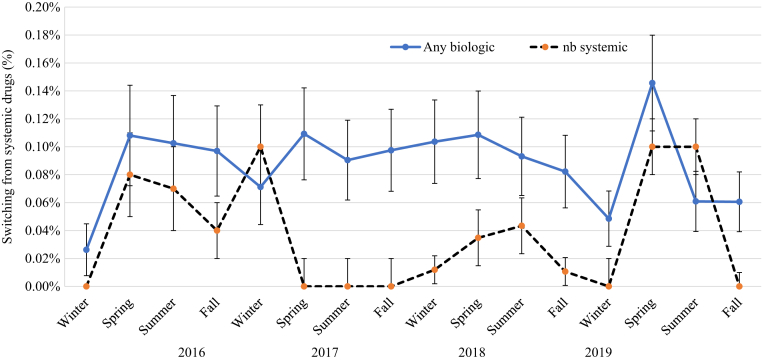
**The impact of season on switching from systemic drugs in patients with psoriasis in 2016‒2019.** nb, nonbiologic.

Figure 11 shows that the incidence of switching was higher for biologics (ranging from 0.03 to 0.15%) than for nonbiologic systemic drugs (ranging from 0 to 0.10%) across all seasons. The switching of nonbiologic drug therapy seemed to be on lack of clear pattern, with a tendency of being lower in winter and higher in spring and summer. The switching of biologics appeared to be lower in winter and higher in spring. Stratified analyses by season did not show clear seasonality, and the incidence of switching was not different by any stratifying variables, except that switching appeared to be higher in PsA than in non-PsA (data not shown). The mean incidence of switching from biologic therapy (95% CI) in 2016‒2019 in winter was 0.07% (95% CI = 0.04‒0.09%) as opposed to 0.12% (95% CI = 0.09‒0.15%) in spring and 0.08% (95% CI = 0.06‒0.11%) in summer and fall (see Table 6). Stratified analysis clearly showed that the mean incidence of switching from biologics appeared highest in spring, except for those aged <20 and ≥70 years. The incidence of switching from TNF-α inhibitors and IL-17 inhibitors appeared to be higher than that of switching from IL-12/IL-23 inhibitors. The results of the meta-regression analysis in Table 7 does not indicate a statistically significant seasonal effect on the incidence of switching for biologics, although the point estimate indicated a trend of higher switching in summer. The highest incidence of switching in spring (see Table 6) supports the peak in the initiation of biologic drugs in spring.

**Table 6:** Incidence (%) and 95% Confidence Intervals of Switching of Biologic Therapy Stratified by Different Factors in Patients with Psoriasis in 2016‒2019

Factors	Winter, 2016‒2019	Spring, 2016‒2019	Summer, 2016‒2019	Fall, 2016‒2019	Mean, 2016–2019
All	0.07 (0.04**‒**0.09)	0.12 (0.09**‒**0.15)	0.08 (0.06**‒**0.11)	0.08 (0.06**‒**0.11)	0.09 (0.06**‒**0.12)
Biologic class					
TNF-α inhibitors	0.07 (0.04**‒**0.10)	0.13 (0.09**‒**0.17)	0.09 (0.05**‒**0.12)	0.08 (0.04**‒**0.11)	0.09 (0.06**‒**0.13)
IL-17 inhibitors	0.13 (0.03**‒**0.23)	0.15 (0.05**‒**0.26)	0.11 (0.03**‒**0.19)	0.13 (0.04**‒**0.21)	0.13 (0.04**‒**0.22)
IL-12/23 inhibitors	0.02 (0.0**‒**0.05)	0.05 (0.01**‒**0.10)	0.04 (0.00**‒**0.08)	0.04 (0.00**‒**0.08)	0.04 (0.00**‒**0.08)
Sex					
Male	0.06 (0.03**‒**0.09)	0.08 (0.05**‒**0.12)	0.09 (0.05**‒**0.13)	0.07 (0.04**‒**0.10)	0.07 (0.04**‒**0.11)
Female	0.08 (0.04**‒**0.12)	0.17 (0.11**‒**0.22)	0.08 (0.04**‒**0.12)	0.10 (0.06**‒**0.15)	0.11 (0.06**‒**0.15)
Age (y)					
<20	0 (0**‒**0)	0.19 (0**‒**0.55)	0.31 (0**‒**0.75)	0 (0**‒**0)	0.13 (0**‒**0.44)
20‒29	0 (0**‒**0)	0.09 (0**‒**0.21)	0 (0**‒**0)	0.04 (0**‒**0.13)	0.03 (0**‒**0.11)
30‒39	0.03 (0**‒**0.08)	0.23 (0.11**‒**0.36)	0.11 (0.03**‒**0.19)	0.11 (0.03**‒**0.19)	0.12 (0.03**‒**0.21)
40‒49	0.08 (0.02**‒**0.14)	0.12 (0.05**‒**0.19)	0.10 (0.04**‒**0.16)	0.07 (0.02**‒**0.12)	0.09 (0.03**‒**0.15)
50-59	0.09 (0.03**‒**0.14)	0.12 (0.06**‒**0.18)	0.06 (0.01**‒**0.10)	0.09 (0.04**‒**0.15)	0.09 (0.04**‒**0.14)
60‒69	0.08 (0.02**‒**0.15)	0.10 (0.03**‒**0.16)	0.08 (0.02**‒**0.14)	0.09 (0.03**‒**0.16)	0.09 (0.03**‒**0.15)
≥70	0.03 (0**‒**0.08)	0 (0**‒**0)	0.12 (0.02**‒**0.23)	0.04 (0.0**‒**0.10)	0.05 (0**‒**0.12)
PsA					
Yes	0.11 (0.06**‒**0.15)	0.17 (0.11**‒**0.22)	0.11 (0.06**‒**0.15)	0.09 (0.05**‒**0.13)	0.12 (0.07**‒**0.16)
No	0.03 (0.01**‒**0.05)	0.09 (0.05**‒**0.12)	0.07 (0.04**‒**0.10)	0.08 (0.05**‒**0.11)	0.07 (0.04**‒**0.10)
Region					
Northeast	0.07 (0**‒**0.16)	0.05 (0**‒**0.12)	0.11 (0.01**‒**0.21)	0.15 (0.04**‒**0.26)	0.10 (0**‒**0.19)
Midwest	0.03 (0**‒**0.06)	0.08 (0.02**‒**0.13)	0.11 (0.05**‒**0.18)	0.12 (0.05**‒**0.18)	0.09 (0.03**‒**0.14)
South	0.08 (0.04**‒**0.12)	0.15 (0.10**‒**0.21)	0.08 (0.04**‒**0.12)	0.07 (0.03**‒**0.10)	0.10 (0.05**‒**0.14)
West	0.06 (0**‒**0.11)	0.12 (0.04**‒**0.21)	0.04 (0**‒**0.08)	0.04 (0**‒**0.08)	0.06 (0.01**‒**0.12)
Latitude					
≥39.85 °N	0.05 (0.01**‒**0.09)	0.06 (0.02**‒**0.10)	0.11 (0.06**‒**0.17)	0.11 (0.06**‒**0.16)	0.09 (0.04**‒**0.13)
<39.85 °N	0.07 (0.04**‒**0.10)	0.15 (0.10**‒**0.19)	0.07 (0.04**‒**0.10)	0.07 (0.04**‒**0.10)	0.09 (0.06**‒**0.12)
Humidity					
≥77.1%	0.06 (0.02**‒**0.10)	0.10 (0.05**‒**0.15)	0.08 (0.04**‒**0.12)	0.09 (0.04**‒**0.13)	0.08 (0.04**‒**0.12)
<77.1%	0.07 (0.04**‒**0.10)	0.13 (0.09**‒**0.18)	0.09 (0.05**‒**0.12)	0.08 (0.05**‒**0.11)	0.09 (0.06**‒**0.13)

Abbreviation: PsA, psoriatic arthritis.

**Table 7:** Relative Risk and 95% CI for Switching of Biologic Therapy: Meta-Regression Analysis Using Robust Variance Estimation Method and Aggregate Data from 16 Seasons in 2016‒2019

Factors	Winter Vs. Spring	Summer Vs. Spring	Fall Vs. Spring
All	0.47 (0.00–49.85)	0.73 (0.42–1.25)	0.71 (0.12–4.29)
Biologic class			
TNF-αi	NA	NA	NA
IL-17i	0.84 (0.31–2.28)	0.81 (0.3–2.19)	0.51 (0.19–1.35)
IL-12/23i	NA	NA	NA
IL-23i	NA	NA	NA
Sex			
Male	0.80 (0.34–1.85)	1.08 (0.44–2.64)	0.78 (0.35–1.75)
Female	0.47 (0.38–0.58)	0.5 (0.45–0.56)	0.63 (0.57–0.71)
Age (y)			
<20	NA	NA	NA
20-29	NA	NA	1.21 (0.55–2.63)
30-39	0.32 (0.11–0.97)	0.46 (0.43–0.49)	0.65 (0.33–1.31)
40–49	0.82 (0.29–2.31)	0.9 (0.28–2.85)	0.62 (0.16–2.31)
50–59	NA	NA	NA
60–69	NA	NA	NA
≥70	NA	NA	NA
PsA			
Yes	NA	NA	NA
No	0.47 (0.09–2.53)	0.82 (0.54–1.22)	0.89 (0.75–1.06)
Region			
Northeast	NA	NA	NA
Midwest	0.56 (0.15–2.02)	NA	NA
South	NA	NA	NA
West	NA	NA	NA
Latitude			
≥39.85 °N	0.65 (0.44–0.95)	1.42 (0.37–5.45)	1.35 (0.77–2.34)
<39.85 °N	0.41 (0.24–0.7)	0.5 (0.5–0.5)	0.45 (0.35–0.59)
Humidity			
≥77.1%	NA	0.81 (0.28–2.35)	0.93 (0.11–8.19)
<77.1%	NA	NA	NA

Abbreviations: CI, confidence interval; IL-17i, IL-17 inhibitor; IL-23i, IL-23 inhibitor; TNFαi, TNF-α inhibitor; NA, not applicable; IL-12/23i, IL-12/23 inhibitor.

Note: Robust variance estimation was used to estimate the covariance matrix of the correlated coefficients in the meta-regression accounting for the clustering within the data due to repeated measures (Fisher et al., 2017). The meta-regression model was fitted with the incidence transformed into a natural logarithmic scale and season as a fixed effect. For IL-17 inhibitors, only the data from 2018 and 2019 are used, to minimize the effect of multiple new products launched in 2015 and 2016.

## Discussion

This study found that the initiation of systemic drugs for psoriasis peaked in spring and then declined in summer and fall. This pattern was consistent for all biologics and nonbiologic systemic drugs and within strata of potentially exacerbating factors for psoriasis. The incidence of initiation of any biologic was much higher in males than in females, highest in those aged 30‒39 years than in other age groups; those with comorbid PsA were more likely to receive systemic drugs. The incidence of any biologic initiation appeared to be higher in the South and Midwest regions, low latitude regions, and low humidity regions than in other regions. In addition, this study found that discontinuation of systemic drugs peaked in winter or summer and that switching from systemic drugs tended to be lower in winter and higher in spring and summer.

No previous studies were identified that evaluated seasonality and environmental factors in relation to initiation and discontinuation of systemic therapies for psoriasis. However, several studies have reported a seasonal relationship to psoriasis in general (Hancox et al., 2004; Harvell and Selig, 2016; Jensen et al., 2022; Kardeş, 2019; Wu et al., 2020). Two studies reported that the frequency of Google search data for the term psoriasis and related terms peaked in the late winter/early spring and troughed in the late summer/early fall (Kardeş, 2019; Wu et al., 2020). Our study found that the initiation of systemic drugs peaked in spring, which was supported by another study (Hancox et al., 2004). Hancox et al. (2004) reported that there is seasonal utilization of dermatologic care in the US and that dermatologic office visits peaked in spring and troughed in fall (33.8 vs. 20.3% of annual visits).

Several studies identified a seasonal association with the worsening of psoriasis symptoms. One study from India reported that 42% of patients with psoriasis worsened in winter versus 8% in summer, whereas 43% improved in the summer, and only 7% improved in winter (Kaur et al., 1997). An online survey of adults with psoriasis from 15 countries reported that 77% of respondents reported seasonal variation of psoriasis exacerbation most notably in winter (67.1%) compared with 23.8% in summer, 7% in spring, and 2.1% in autumn (Ferguson et al., 2021). A nationwide survey of over 12,000 patients with psoriasis in China found that season change was the most frequently reported cause of relapse or aggravation (60.2%). Nearly half of reports about the weather as an aggravating factor were related to the winter season (48.8%), followed by spring (23.1%), autumn (17.1%), and summer (8.4%) (Chen et al., 2017). A retrospective study of 2,270 patients with psoriasis in China found that a total of 53.2% reported the seasonal pattern of disease, with psoriasis exacerbation in fall/winter (Zheng et al., 2021). Oral or biologic treatment may be initiated if psoriasis is too extensive for topical therapy or refractory to topical therapy and phototherapy (Menter et al., 2009). The winter season, with shorter daylight than other seasons, is an aggravating factor for patients with psoriasis (Chen et al., 2017). Our study found the highest initiation rate in the South region. South region in the US has been associated with the highest proportion of patients with obesity and very severe psoriasis (body surface area > 20%) in the US Corona Psoriasis Registry (Enos et al., 2021), which reported that psoriasis severity and comorbidities differed among US geographic regions (Enos et al., 2021).

Our finding of seasonal variation in the initiation of systemic drugs for psoriasis has not been reported in the literature and has important implications. A recent systematic review of 13 studies reported that about 50% of patients with psoriasis were stable and showed no seasonal differences between seasons and that approximately 30% improved in summer and 20% performed better in winter (Jensen et al., 2022). Guidelines on the management of psoriasis are suggested to add seasonality so that treatment and patient education may be considered to prevent disease worsening. Although the relative seasonal change for the initiation of systemic drugs appeared small in this study (0.1‒0.4% for biologics and 0.1‒0.3% for nonbiologic systemic drugs), the mean reduction for biologic initiation from 1.28% in spring to 1.11% in summer could lead to an estimated decrease in the number of biologic initiators for over 14,280 patients, considering that there are over 7.5 million adults and 0.9 million children with psoriasis in the US (Armstrong et al., 2021; Paller et al., 2018). Therefore, although the relative seasonal change is small, the absolute seasonal change has clinical significance because the findings provide evidence for healthcare resource planning in psoriasis management. In addition, we found that the mean incidence of biologic switching appeared to be highest in spring. This change in systemic drug use may not reflect the disease severity because patients’ behaviors around wanting to be free of psoriasis and be able to wear short sleeves/swim in warmer months may impact their use of systemic drugs. However, the initiation of systemic drugs in the study was determined on the basis of the date of systemic drug dispense or administration. It is typically related to a clinical visit or a medical claim with a psoriasis diagnosis. The timing of initiation of a new systemic drug may indicate a potential psoriasis flare for the patient. Whether a higher proportion of patients discontinued biologics owing to psoriasis improvement in the summer is unknown. Thus, the findings from this study may be used for hypothesis generating, and further patient-level‒based research is needed.

Our finding that the incidence of the initiation of biologic drug peaks in spring is supported by the higher incidence of discontinuation and switching from nonbiologic systemic drugs in spring and the higher incidence of switching from biologics in spring. Although the 95% CIs overlapped, the mean increase for biologic switching from 0.07% in winter to 0.12% in spring would result in an estimated increase in the number of biologic switchers for over 840 patients, considering a US psoriasis population of approximately 8.4 million (Armstrong et al., 2021; Paller et al., 2018) and that about 20% are moderate or severe (Menter et al., 2008). We found that the mean incidence of biologics discontinuation appeared to be highest in summer, except for those aged ≥60 years and those with PsA. Switching or discontinuation from biologics could be due to primary ineffectiveness, secondary loss of response, side effects, patient preferences, comorbidities, and economic burden (Bayaraa and Imafuku, 2019; Florek et al., 2018). Treatment switch is frequent in psoriasis, with 50% of traditional systemic‒treated patients switching to a biologic; the age of these patients tended to be younger. Conversely, 25% of the biologic group transitioned to traditional oral systemic therapy; these patients tended to be older and have a longer duration of disease (Tabolli et al., 2015).

This study found that the initiation of any biologics varied by patient characteristics. Patients’ preferences for certain treatments may depend on age, sex, comorbidities, disease duration, and prior treatments (Florek et al., 2018).

The higher incidence of initiation of any biologics in males than in females may reflect patient preference and that men with psoriasis are more likely to have severe psoriasis than women (Florek et al., 2018; Hägg et al., 2013). In addition, men with psoriasis have been found to be more concerned about efficacy than women (Kromer et al., 2015). The finding that the highest incidence of initiation of systemic drugs occurred in patients aged 30‒39 years is also supported by the literature (Bayaraa and Imafuku, 2019; Florek et al., 2018; Geale et al., 2016; Gorelick et al., 2019). Psoriasis impacts the patients’ self-esteem and QOL (Kubanov et al., 2018; Nazik et al., 2017; Pariser et al., 2016). At least 90% of young patients with psoriasis value clear skin, sustained response, and rapid onset of action (Gorelick et al., 2019). As people age, patients with psoriasis have fewer opportunities to access biologic medications (Geale et al., 2016).

This study has several strengths. First, the exposure season is clearly defined. This study assessed treatment patterns using data from all the four years, and similar patterns were observed. In addition, the seasonal pattern in the initiation of systemic drugs is supported by the discontinuation and switching data. Second, effect modifiers were stratified to assess the association between seasons and treatment patterns of systemic drugs, and similar patterns held after stratification. Third, study findings may not only inform healthcare providers and patients in their decision making in patient disease management but also generate, to our knowledge, previously unreported research ideas.

A few limitations need to be mentioned. First, although a clear association has been identified between seasons and initiation, discontinuation, and switching of systemic drugs in patients with psoriasis, no causal relationship can be made. Second, patients with rheumatoid arthritis, ankylosing spondylitis, inflammatory bowel disease, and other conditions for which certain biologics may be indicated were not excluded from the study. Therefore, there might be some misclassification in the initiation, discontinuation, and switching of certain biologics. However, the impact could be minimal given that the prevalence of these conditions is less common. Third, the association between season and outcomes was not assessed in a mixed-effect regression model, where the season can be a fixed effect, and a patient can be a random effect to derive the seasonal percentages while accounting for the correlation among repeated measures. Future studies may be conducted to assess whether the findings from this study are true in patient-level drug utilization studies in psoriasis population (which will allow patients to be followed up without interruption of season end) and whether season is an independent factor to predict change in systemic therapy while controlling for other factors. Finally, patients in this database are covered by commercial insurance. Healthcare insurance coverage may affect treatment patterns as well (Armstrong et al., 2017). The study findings may not be generalizable to those without healthcare insurance or covered by different healthcare insurance.

In summary, a seasonal pattern of initiation of systemic drug therapies for psoriasis is identified in this study. However, this finding needs to be interpreted with caution because initiation of a systemic therapy does not indicate a reactive response to a loss of disease control and increased symptoms. Discontinuing ineffective drugs and switching to alternative systemic drugs among individuals who use medications in reaction to psoriasis symptoms might be a key component in reducing the risk of psoriasis worsening. Future research may focus on specific systemic drug survival rates and switching patterns among patients with certain comorbidities.

## Materials and Methods

### Study design

The study is a retrospective ecological study of individuals with psoriasis identified in the Optum Clinformatics Data Mart. Two cohorts were generated for each season of 2016‒2019: patients with psoriasis who were eligible for initiating a systemic drug and those with psoriasis who were prevalent users of a systemic drug. The outcomes were the incidence of initiation of a systemic drug in each season and the incidence of switching from or discontinuing a systemic drug. They were assessed separately for each season. Different cohorts were used to assess the association of season and other factors and the use of systemic drugs for psoriasis.

### Study setting

The Optum Clinformatics Data Mart is commercially available in the US. It contains longitudinal commercial and Medicare Advantage health plan data from 50 US states since 2000. The claims data include member eligibility, medical and pharmacy claims, and inpatient confinements. It covered approximately 16 million annual lives associated with United Healthcare plans for over 80 million unique lives. On the basis of the Health Insurance Portability and Accountability Act in the US, all personal identifiers for patients are anonymized by applying specific algorithms. For example, patient names, social security numbers, and five-digit zip codes were removed from the databases. The date of birth was converted to the year of birth before data release. Thus, institutional review board review was unnecessary.

### Study population

The study population included patients with psoriasis, defined as those with a diagnosis of psoriasis (International Classification of Diseases [ICD], Ninth Revision, Clinical Modification = 696.1; ICD, Tenth Revision, Clinical Modification = L40.0) at any time in their medical history before December 31, 2019 and were enrolled in Optum Clinformatics Data Mart between January 1, 2016 and December 31, 2019. Two diagnosis codes for psoriasis were not required because (i) a patient may initiate a new treatment after one diagnosis of psoriasis and requiring two diagnoses codes would arbitrarily ignore the initiating date of the treatment between the two diagnoses codes, which made the initiating date of the treatment inaccurate, and (ii) for patients who were eligible to discontinue a systemic drug or eligible to switch from a systemic drug, one diagnosis of psoriasis plus a prescription claim for a systemic drug were used to identify psoriasis cohort, which has been reported to have a positive predictive value of 78.4% (Lee et al., 2021).

Denominator cohorts consisted of patients with psoriasis who were eligible for initiating, switching from, or discontinuing a systemic drug and were assessed separately by season. The denominator cohorts were determined on the first date of each season (cohort entry date [CED]) using treatment data 6 months before the CED. A total of 16 denominator cohorts were created for patients with psoriasis who were eligible for initiating a nonbiologic systemic drug during the study period.

#### Eligibility of denominator cohorts

**Figure 12:**
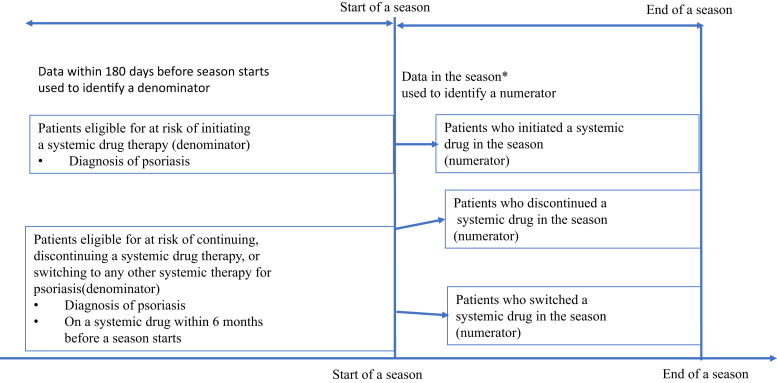
**Denominators and numerators for initiation, discontinuation, and switching.** The asterisk (∗) indicates that the season for winter 2016 used data from January to February 2016.

Patients were eligible for initiating a drug if they had psoriasis and were at risk of initiating the systemic drug. For example, only patients who had not initiated the drug of interest in the 180 days before January 2016 were considered at risk of initiating the drug on January 1, 2016. Person-time at risk referred to person-time in 2016‒2019 that a patient contributed after a psoriasis diagnosis and before the systemic drug initiation. Patients who had a psoriasis diagnosis and continuously used a systemic agent were eligible for discontinuing or switching from the systemic drug. Only person-time that met these criteria was considered at risk of discontinuation or switching. In evaluating initiation, discontinuation, and switching of systemic drugs, a 30-day gap was allowed between prescription refills. If the same drug has multiple prescriptions with overlapping periods, the treatment duration was the sum of those periods. An at-risk patient was followed until the earliest occurrence of initiating, switching from, or discontinuing a systemic agent; death; disenrollment; end of the study; and ceased contributing person-time. Numerators cohorts came from the denominator cohorts and were patients who initiated, switched from, or discontinued a systemic drug during the season of interest and were also assessed separately for the season of interest. The numerator cohorts were determined using treatment data at least 6 months before the season and treatment data during each season in 2016‒2019. Figure 12 presents how denominators and numerators for initiation, discontinuation, and switching are estimated in each season.

### Exposures

The exposure was periods of seasons and defined as winter (December–February), spring (March–May), summer (June–August), and fall (September–November) (Trenberth, 1983) in 2016‒2019; except for winter 2016, only January and February data were used.

### Outcomes

The outcomes were incidence of initiating, switching from, and discontinuing a systemic drug, first assessed for each drug of interest and then grouped by drug classes.

Specifically, initiation of a systemic drug was defined as the first date of that drug dispensing or administration in the season under study. To determine initiation, the use of systemic medications was assessed using data 180 days before CED. Switching from one systemic drug to another was defined as the first date for a prescription stream for an alternative drug that occurred within 30 days before a patient exhausted or discontinued his initial therapy. Discontinuation of a systemic drug was defined as the last day of a therapy stream, which required at least 30 days free of any biologic or nonbiologic therapy after the end of the therapy stream. For all these outcomes, the first event for drug initiation, discontinuation, and switching was assessed in each season, ranging from 90 to 92 days. It is less likely that a clinician would change a patient’s systemic therapy two times in such a short time window. Torres et al. (2021) reported that at 12 months, IL-23 inhibitors had over 90% cumulative probability of drug survival, whereas an IL-17 inhibitor had the lowest cumulative probability of 85.5% for drug survival. However, patients who received the second or third biologic and so on for 180 days in the following seasons would qualify to be assessed for further initiation, switching, or discontinuation.

The systemic drug classes assessed comprised TNF-α inhibitors, IL-12 and IL-23 inhibitors, IL-17 inhibitors, IL-23 inhibitors, any biologics, and nonbiologic systemic immunosuppressants. Table 8 presents the generic names for systemic medications and their use frequencies and prescription length. The number of days covered with each biologic was captured on the basis of its mode of administration. Self-administered biologics dispensed at the pharmacy were identified from prescription claims using National Drug Codes, and days of supply were used to calculate the number of days covered by each prescription. Biologics that required infusion under the supervision of medical professionals were identified from medical claims using the Healthcare Common Procedure Coding System codes. The administration date and the assigned days’ supply for each administration based on recommended dosage regimen were used to calculate the estimated medication end date. For biologics that were found in both prescriptions claims and medical claims, the medication end date was estimated using the prescription fill date and days of supply or administration date and assigned days of supply.

**Table 8:** Biologic and Nonbiologic Systemic Treatment in the Study

Type of Treatment	Approval Date	Drug Substance	Mechanism of Action	Half-Life (d)	Maintenance Dosing Interval and Route of Administration	Standard Duration of Prescriptions (Days of Service)
Biologic: IL-23 inhibitors	4/23/2019	Risankizumab	IL-23A	28	150 mg every 12 weeks, sc	84
	7/13/2017	Guselkumab	IL-23A	18	100 mg every 8 weeks, sc	56
	3/21/2018	Tildrakizumab	IL-23A	23	100 mg every 12 weeks, sc	84
Biologic: IL-17 inhibitors	1/21/2015	Secukinumab	IL-17A	27	300 mg every 4 weeks, sc	28
	3/22/2016	Ixekizumab	IL-17A	13	80 mg every 4 weeks, sc	28
	2/15/2017	Brodalumab	IL-17RA	11	210 mg every 2 weeks, sc	14
Biologic biosimilars	4/5/2016	IFX-DYYB	TNF-α inhibitor	9.5	5 mg/kg every 8 weeks, iv	56
	4/21/2017	IFX-ABDB	TNF-α inhibitor	9.5	5 mg/kg every 8 weeks, iv	56
	12/13/2017	IFX-QBTX	TNF-α inhibitor	9.5	5 mg/kg every 8 weeks, iv	56
	8/30/2016	ETA-SZZS	TNF-α inhibitor	3	50 mg once a week, sc	7
Biologic: IL-12/23 inhibitor	9/25/2009	Ustekinumab	IL-12/IL-23	21	45/90 mg every 12 weeks, sc/iv	84^1^
Biologic: TNF-α inhibitors	4/30/2004	Etanercept	TNF-α inhibitor	3	50 mg once a week, sc	7
	9/26/2006	Infliximab	TNF-α inhibitor	9.5	5 mg/kg every 8 weeks, iv	56
	1/18/2008	Adalimumab	TNF-α inhibitor	14	40 mg every 2 weeks, sc	14
	5/29/2018	Certolizumab	TNF-α inhibitor	14	200 mg every 2 weeks, sc	14
Nonbiologic systemic drugs	NA	Acitretin	Retinoid/unknown	2	daily	31
	NA	Cyclosporine	Immunosuppressive	8.4 hours	2.5‒4.0 mg/kg/day for 6‒16 weeks, oral	31
	NA	Methotrexate	Antimetabolite	6.5 hours	10‒25 mg per week, po, sc	7
	9/23/2014	Apremilast	PDE-4 inhibitor		30 g twice daily, po	31

Abbreviations: iv, intravenous; NA, not applicable; PDE-4, phosphodiesterase-4; po, oral administration; sc, subcutaneous.

Note: All prescription claims were allocated a minimum duration of 31 days.

1 Days of service was estimated based on subcutaneous administration in psoriasis.

### Covariates

Age on CED, sex, and region of residence were demographic variables. Patient-level data included sex (male vs. female); age subgroups (<20, 20‒29, 30‒39, 40‒49, 50‒59, 60‒69, and ≥70 years); and status of comorbid PsA, which was identified on the basis of one diagnosis code of ICD (ICD, Ninth Revision, Clinical Modification = 696.0 or ICD, Tenth Revision, Clinical Modification = L40.50, L40.51, L40.52, L40.53, L40.54, L40.59) using data before CED. Prescribers were classified as dermatologists and nondermatologists (physician assistant, general practitioner/internist, rheumatologist, and others), identified on the basis of the provider information on medical claims and the National Uniform Claim Committee provider taxonomy codes in the provider file. On the basis of the state a patient resided, US geographic regions (Northeast, Midwest, West, and South), low and high latitude (<39.85 °N vs. ≥39.85 °N), and humidity (<77.1% vs. ≥77.1%) were derived on the basis of the median of all states (see Table 9). These variables were considered possible effect modifiers.

**Table 9:** Classification of Region, Latitude, and Humidity in the US

Factors	Category	US States
Region^1^	Northeast	Connecticut, Maine, Massachusetts, New Hampshire, Rhode Island, Vermont, New Jersey, New York, Pennsylvania
	Midwest	Indiana, Illinois, Michigan, Ohio, Wisconsin, Iowa, Nebraska, Kansas, North Dakota, Minnesota, South Dakota, Missouri
	South	Delaware, District of Columbia, Florida, Georgia, Maryland, North Carolina, South Carolina, Virginia, West Virginia, Alabama, Kentucky, Mississippi, Tennessee, Arkansas, Louisiana, Oklahoma, Texas
	West	Arizona, Colorado, Idaho, New Mexico, Montana, Utah, Nevada, Wyoming, Alaska, California, Hawaii, Oregon, Washington
Latitude^2^	Latitude <39.85 °N	West Virginia, Texas, Kansas, Mississippi, Delaware, Arkansas, Missouri State, Florida, Georgia, Hawaii, Tennessee, Virginia, New Jersey, Kentucky, Oklahoma Utah, Colorado, Alabama, New Mexico, South Carolina Arizona, Maryland, California, North Carolina, Louisiana
	Latitude ≥39.85 °N	Wisconsin, Vermont, South Dakota, Rhode Island, Oregon, New York, New Hampshire, Nebraska, Illinois, Connecticut, Indiana, Nevada, Maine, Michigan Alaska, North Dakota, Minnesota, Montana, Washington, Ohio, Iowa, Pennsylvania, Massachusetts Idaho, Wyoming
Humidity^3^	Humidity <77.1%	Florida, North Carolina, Texas, Illinois, Arkansas, Vermont, Virginia, Oklahoma, New Mexico, Alabama, Kentucky, Indiana, Georgia, Mississippi, Louisiana, New York, Tennessee, Massachusetts, Utah, Michigan, Hawaii, South Carolina, Maryland, Delaware, New Jersey
	Humidity ≥77.1%	Iowa, New Hampshire, Alaska, Maine, North Dakota, Minnesota, South Dakota, Montana, California, Colorado, Oregon, Idaho, Wyoming, Arizona, Kansas, Connecticut, Washington, Nebraska, West Virginia, Nevada, Pennsylvania, Missouri, Ohio, District of Columbia, Rhode Island, Wisconsin

Abbreviation: US, United States.

1 Region was classified on the basis of the US census region (United States Census Bureau, 2013).

2 Latitude was classified on the basis of the median latitude of US states.

3 Humidity was classified on the basis of the median humidity of US states (USA.com, 2014).

### Data analysis

A patient flow chart was generated for each study cohort. Patient demographics were summarized with descriptive statistics. The incidence of initiating a systemic drug was calculated using the number of patients who initiated a systemic drug during the season divided by the number of patients with psoriasis who were eligible for initiating a systemic drug on CED. Similarly, the incidence of switching from or discontinuing a systemic drug was calculated using the number of patients who switched from or discontinued a systemic drug during the season divided by the number of patients with psoriasis who used the systemic drug before CED. The incidence of initiation, discontinuation, and switching and 95% CIs were calculated separately for all seasons in 2016‒2019. Of note, winter 2016 was from January 1, 2016 to February 29, 2016; thus, the incidence for winter 2016 needs to be interpreted with caution. In addition, the mean incidence and 95% CI for winter through the fall season in 2016‒2019 were also calculated. To determine a seasonal trend, we compared the 95% CIs for the incidence of initiation, discontinuation, and switching for each season. If the 95% CIs among seasons do not overlap, then the two incidences are considered statistically different. To account for the clustering within the data due to repeated measures, meta-regression analyses with robust variance estimation were conducted for biologics. The robust variance estimation considers the covariances between outcomes from different studies and provides an estimation of covariance matrix. The estimates from each season are considered separate studies for initiation, discontinuation, and switching. Therefore, data from 16 studies (seasons) were used in each meta-regression analysis. Meta-regression models were fitted with all the incidences transformed to natural logarithmic scale and season as a fixed effect for the incidence of initiation, discontinuation, and switching for all biologics and stratified by patient characteristics, region, latitude, and humidity. Meta-regression analysis results were presented in Tables 3, 5, and 7. In addition, line graphics were used to show the seasonal trend for systemic drugs overall and the seasonal trend stratified by each covariate. Finally, to show clinical significance, the change in the number of biologic initiators from spring to summer in 1 year was estimated on the basis of the absolute difference in the mean incidence of biologic initiation between spring and summer in 2016‒2019 and estimated psoriasis population in the US (Armstrong et al., 2021; Paller et al., 2018); the change in the number of biologic switchers from winter to spring in 1 year was estimated on the basis of the absolute difference in the mean incidence of biologic switching between winter and spring in 2016‒2019 and estimated moderate-to-severe psoriasis population in the US (Armstrong et al., 2021; Menter et al., 2008; Paller et al., 2018).

To assess the impact of season on the initiation, switching, and discontinuation of systemic drugs, analyses were performed in the AETION Evident Platform, a cloud-based scientifically validated software that transforms real-world data into transparent, reliable, and replicable real-world evidence (Folkerts et al., 2020; Garry et al., 2019; Seesaghur et al., 2021). All analyses were stratified by possible effect modifiers of the association between season and initiation, discontinuation, and switching of systemic drugs. No patient-level datasets can be downloaded from the seasonality analysis module of the AETION Evident Platform so mixed-effect regression analysis of the patient-level data is not feasible. However, Rstudio, version 3.6.0, was used for the entry of data from 16 seasons for biologic initiation, discontinuation, and switching. Robumeta package in R was used for meta-regression analyses (Fisher et al., 2017).

### Data availability statement

No large datasets were generated or analyzed during this study. Minimal datasets necessary to interpret and/or replicate data in this paper are available on request to the corresponding author.

## ORCIDs

Huifang Liang: http://orcid.org/0000-0002-4145-4784

Brenna Kirk: http://orcid.org/0000-0003-1579-3499

Jennifer M. Polinski: http://orcid.org/0000-0002-4571-1434

Xiaomeng Yue: http://orcid.org/0000-0002-4418-7079

Ryan D. Kilpatrick: http://orcid.org/0000-0003-1388-3669

Joel M. Gelfand: http://orcid.org/0000-0003-3480-2661

## Conflict of Interest

HL, XY, and RDK are AbbVie employees and may own AbbVie stock/options. BK and JMP are AETION employees and may own AETION stock/options. JMG served as a consultant for AbbVie, Bristol-Myers Squibb, Boehringer Ingelheim, GlaxoSmithKline, Happify, Janssen Biologics, Lilly (DMC), Leo, Novartis, Pfizer, Regeneron, UCB (Data Safety and Monitoring Board), Neuroderm (Data Safety and Monitoring Board), Sanofi, and Mindera Dx, receiving honoraria; in addition, he receives research grants (to the Trustees of the University of Pennsylvania, Philadelphia, PA) from AbbVie, Amgen, Boehringer Ingelheim, Janssen, Novartis, Sanofi, Celgene, OrthoDermatologics, and Pfizer, and he has received payment for continuing medical education work related to psoriasis that was supported indirectly by Eli Lilly and Company and Ortho Dermatologics. In addition, JMG is a copatent holder of resiquimod for the treatment of cutaneous T-cell lymphoma, and he is a deputy editor for the *Journal of Investigative Dermatology*, receiving honoraria from the Society for Investigative Dermatology; is Chief Medical Editor for Healio Psoriatic Disease (receiving honoraria); and is a member of the Board of Directors for the International Psoriasis Council, receiving no honoraria.

## References

[bib1] Armstrong A.W., Koning J.W., Rowse S., Tan H., Mamolo C., Kaur M. (2017). Under-treatment of patients with moderate to severe psoriasis in the United States: analysis of medication usage with health plan data. Dermatol Ther (Heidelb).

[bib2] Armstrong A.W., Mehta M.D., Schupp C.W., Gondo G.C., Bell S.J., Griffiths C.E.M. (2021). Psoriasis prevalence in adults in the United States. JAMA Dermatol.

[bib3] Aune D., Snekvik I., Schlesinger S., Norat T., Riboli E., Vatten L.J. (2018). Body mass index, abdominal fatness, weight gain and the risk of psoriasis: a systematic review and dose-response meta-analysis of prospective studies. Eur J Epidemiol.

[bib4] Bayaraa B., Imafuku S. (2019). Sustainability and switching of biologics for psoriasis and psoriatic arthritis at Fukuoka University Psoriasis Registry. J Dermatol.

[bib6] Brezinski E.A., Dhillon J.S., Armstrong A.W. (2015). Economic burden of psoriasis in the United States: a systematic review. JAMA Dermatol.

[bib7] Chandran V., Raychaudhuri S.P. (2010). Geoepidemiology and environmental factors of psoriasis and psoriatic arthritis. J Autoimmun.

[bib8] Chen K., Wang G., Jin H., Xu J., Zhu X., Zheng M. (2017). Clinic characteristics of psoriasis in China: a nationwide survey in over 12000 patients. Oncotarget.

[bib9] Crown W.H., Bresnahan B.W., Orsini L.S., Kennedy S., Leonardi C. (2004). The burden of illness associated with psoriasis: cost of treatment with systemic therapy and phototherapy in the US. Curr Med Res Opin.

[bib11] Dubertret L., Mrowietz U., Ranki A., van de Kerkhof P.C.M., Chimenti S., Lotti T. (2006). European patient perspectives on the impact of psoriasis: the EUROPSO patient membership survey. Br J Dermatol.

[bib12] Egeberg A. (2016). Psoriasis and comorbidities. Epidemiological studies. Dan Med J.

[bib13] Enos C.W., O'Connell K.A., Harrison R.W., McLean R.R., Dube B., Van Voorhees A.S. (2021). Psoriasis severity, comorbidities, and treatment response differ among geographic regions in the united states. JID Innov.

[bib14] Fahey M.C., Klesges R.C., Kocak M., Talcott G.W., Krukowski R.A. (2020). Seasonal fluctuations in weight and self-weighing behavior among adults in a behavioral weight loss intervention. Eat Weight Disord.

[bib15] Farber E.M., Nall L. (1984). An appraisal of measures to prevent and control psoriasis. J Am Acad Dermatol.

[bib16] Feldman S.R., Tian H., Gilloteau I., Mollon P., Shu M. (2017). Economic burden of comorbidities in psoriasis patients in the United States: results from a retrospective U.S. database. BMC Health Serv Res.

[bib17] Ferguson F.J., Lada G., Hunter H.J.A., Bundy C., Henry A.L., Griffiths C.E.M. (2021). Diurnal and seasonal variation in psoriasis symptoms. J Eur Acad Dermatol Venereol.

[bib18] Fisher Z., Tipton E., Hou Z. robumeta: robust variance meta-regression; 2017. https://cran.r-project.org/web/packages/robumeta/index.html.

[bib19] Florek A.G., Wang C.J., Armstrong A.W. (2018). Treatment preferences and treatment satisfaction among psoriasis patients: a systematic review. Arch Dermatol Res.

[bib20] Folkerts K., Petruski-Ivleva N., Kelly A., Fried L., Blankenburg M., Gay A. (2020). Annual health care resource utilization and cost among type 2 diabetes patients with newly recognized chronic kidney disease within a large U.S. administrative claims database. J Manag Care Spec Pharm.

[bib21] Garry E.M., Schneeweiss S., Eapen S., Petruski-Ivleva N., Cheever E., Murk W. (2019). Actionable real-world evidence to improve health outcomes and reduce medical spending among risk-stratified patients with diabetes. J Manag Care Spec Pharm.

[bib22] Geale K., Henriksson M., Schmitt-Egenolf M., Evaluating equality in psoriasis healthcare: a cohort study of the impact of age on prescription of biologics (2016). Br J Dermatol.

[bib23] Gorelick J., Shrom D., Sikand K., Renda L., Burge R., Dworkin C. (2019). Understanding treatment preferences in patients with moderate to severe plaque psoriasis in the USA: results from a cross-sectional patient survey. Dermatol Ther (Heidelb).

[bib24] Gutierrez E., Sanmartino C., Carrera O., Fraga A., Arce C. (2017). Psoriasis: latitude does make a difference. J Am Acad Dermatol.

[bib25] Hägg D., Eriksson M., Sundström A., Schmitt-Egenolf M. (2013). The higher proportion of men with psoriasis treated with biologics may be explained by more severe disease in men. PLoS One.

[bib26] Hancox J.G., Sheridan S.C., Feldman S.R., Fleischer A.B. (2004). Seasonal variation of dermatologic disease in the USA: a study of office visits from 1990 to 1998. Int J Dermatol.

[bib27] Harvell J.D., Selig D.J. (2016). Seasonal variations in dermatologic and dermatopathologic diagnoses: a retrospective 15-year analysis of dermatopathologic data. Int J Dermatol.

[bib28] Jensen K.K., Serup J., Alsing K.K. (2022). Psoriasis and seasonal variation: a systematic review on reports from Northern and Central Europe-little overall variation but distinctive subsets with improvement in summer or wintertime. Skin Res Technol.

[bib29] Kardeş S. (2019). Seasonal variation in the internet searches for psoriasis. Arch Dermatol Res.

[bib30] Kaur I., Handa S., Kumar B. (1997). Natural history of psoriasis: a study from the Indian subcontinent. J Dermatol.

[bib31] Korman N.J., Zhao Y., Pike J., Roberts J. (2016). Relationship between psoriasis severity, clinical symptoms, quality of life and work productivity among patients in the USA. Clin Exp Dermatol.

[bib32] Korman N.J., Zhao Y., Pike J., Roberts J., Sullivan E. (2015). Increased severity of itching, pain, and scaling in psoriasis patients is associated with increased disease severity, reduced quality of life, and reduced work productivity. Dermatol Online J.

[bib33] Kromer C., Schaarschmidt M.L., Schmieder A., Herr R., Goerdt S., Peitsch W.K. (2015). Patient preferences for treatment of psoriasis with BioLogicals: a discrete choice experiment. PLoS One.

[bib34] Kubanov A.A., Bakulev A.L., Fitileva T.V., Novoderezhkina E., Gilloteau I., Tian H. (2018). Disease burden and treatment patterns of psoriasis in Russia: a real-world patient and dermatologist survey. Dermatol Ther (Heidelb).

[bib35] Lee H., He M., Cho S.K., Bessette L., Tong A.Y., Merola J.F. (2021). Validation of claims-based algorithms to identify patients with psoriasis. Pharmacoepidemiol Drug Saf.

[bib36] Menter A., Gottlieb A., Feldman S.R., Van Voorhees A.S., Leonardi C.L., Gordon K.B. (2008). Guidelines of care for the management of psoriasis and psoriatic arthritis: Section 1. Overview of psoriasis and guidelines of care for the treatment of psoriasis with biologics. J Am Acad Dermatol.

[bib37] Menter A., Korman N.J., Elmets C.A., Feldman S.R., Gelfand J.M., Gordon K.B. (2009). Guidelines of care for the management of psoriasis and psoriatic arthritis: section 4. Guidelines of care for the management and treatment of psoriasis with traditional systemic agents. J Am Acad Dermatol.

[bib38] Momperousse D., Delnevo C.D., Lewis M.J. (2007). Exploring the seasonality of cigarette-smoking behaviour. Tob Control.

[bib39] Nazik H., Nazik S., Gul F.C. (2017). Body image, self-esteem, and quality of life in patients with psoriasis. Indian Dermatol Online J.

[bib40] Nguyen K.B., Read C., Wu K.K., Armstrong A.W. (2020). Where you live matters: regional differences in health care resource use for psoriasis in the United States. J Am Acad Dermatol.

[bib41] Paller A.S., Singh R., Cloutier M., Gauthier-Loiselle M., Emond B., Guérin A. (2018). Prevalence of psoriasis in children and adolescents in the United States: a claims-based analysis. J Drugs Dermatol.

[bib42] Pariser D., Schenkel B., Carter C., Farahi K., Brown T.M., Ellis C.N. (2016). A multicenter, non-interventional study to evaluate patient-reported experiences of living with psoriasis. J Dermatolog Treat.

[bib43] Rachakonda T.D., Schupp C.W., Armstrong A.W. (2014). Psoriasis prevalence among adults in the United States. J Am Acad Dermatol.

[bib44] Seesaghur A., Petruski-Ivleva N., Banks V., Wang J.R., Mattox P., Hoeben E. (2021). Real-world reproducibility study characterizing patients newly diagnosed with multiple myeloma using Clinical Practice Research Datalink, a UK-based electronic health records database. Pharmacoepidemiol Drug Saf.

[bib45] Skudutyte-Rysstad R., Slevolden E.M., Hansen B.F., Sandvik L., Preus H.R. (2014). Association between moderate to severe psoriasis and periodontitis in a Scandinavian population. BMC Oral Health.

[bib46] Springate D.A., Parisi R., Kontopantelis E., Reeves D., Griffiths C.E., Ashcroft D.M., Incidence, prevalence and mortality of patients with psoriasis: a U.K. population-based cohort study (2017). Br J Dermatol.

[bib47] Tabolli S., Giannantoni P., Paradisi A., Abeni D. (2015). The 'switcher' patient profile in psoriasis treatment: from traditional to biological and from biological to traditional systemic drugs. Br J Dermatol.

[bib48] Takeshita J., Grewal S., Langan S.M., Mehta N.N., Ogdie A., Van Voorhees A.S. (2017). Psoriasis and comorbid diseases: epidemiology. J Am Acad Dermatol.

[bib49] Torres T., Puig L., Vender R., Lynde C., Piaserico S., Carrascosa J.M., Drug survival of IL-12/23, IL-17 and IL-23 inhibitors for psoriasis treatment: a retrospective multi-country, multicentric cohort study (2021). Am J Clin Dermatol.

[bib50] Trenberth K.E. (1983). What are the seasons?. Bull Amer Meteor Soc.

[bib51] United States Census Bureau Census Bureau regions and divisions with states FIPS codes. https://www2.census.gov/geo/pdfs/maps-data/maps/reference/us_regdiv.pdf.

[bib52] USA.com U.S. average humidity state rank - American Community Survey 2010-2014. https://www.usa.com/rank/us%13average-humidity%13state-rank.htm.

[bib56] Villacorta R, Teeple A, Lee S, Fakharzadeh S, Lucas J, McElligott S. (2020). A multinational assessment of work-related productivity loss and indirect costs from a survey of patients with psoriasis. Br J Dermatol.

[bib53] Wolk K., Mallbris L., Larsson P., Rosenblad A., Vingård E., Ståhle M. (2009). Excessive body weight and smoking associates with a high risk of onset of plaque psoriasis. Acta Derm Venereol.

[bib54] Wu Q., Xu Z., Dan Y.L., Zhao C.N., Mao Y.M., Liu L.N. (2020). Seasonality and global public interest in psoriasis: an infodemiology study. Postgrad Med J.

[bib55] Zheng X., Wang Q., Luo Y., Lu W., Jin L., Chen M. (2021). Seasonal variation of psoriasis and its impact in the therapeutic management: a retrospective study on Chinese patients. Clin Cosmet Investig Dermatol.

